# Glycine administration attenuates progression of dystrophic pathology in prednisolone-treated dystrophin/utrophin null mice

**DOI:** 10.1038/s41598-019-49140-x

**Published:** 2019-09-10

**Authors:** Daniel J. Ham, Anastasia Gardner, Tahnee L. Kennedy, Jennifer Trieu, Timur Naim, Annabel Chee, Francesca M. Alves, Marissa K. Caldow, Gordon S. Lynch, René Koopman

**Affiliations:** 1 Centre for Muscle Research, Department of Physiology, The University of Melbourne, Melbourne, Australia; 2 Present Address: Biozentrum, University of Basel, Klinglebergstrasse 50/70, CH-4056 Basel, Switzerland; 3 Present Address: Oxford Neuromuscular Centre, Department of Physiology, Anatomy and Genetics, University of Oxford, Oxford, OX1 3PT UK

**Keywords:** Homeostasis, Preclinical research

## Abstract

Duchenne muscular dystrophy (DMD) is an X-linked genetic disease characterized by progressive muscle wasting and weakness and premature death. Glucocorticoids (e.g. prednisolone) remain the only drugs with a favorable impact on DMD patients, but not without side effects. We have demonstrated that glycine preserves muscle in various wasting models. Since glycine effectively suppresses the activity of pro-inflammatory macrophages, we investigated the potential of glycine treatment to ameliorate the dystrophic pathology. Dystrophic *mdx* and dystrophin-utrophin null (*dko*) mice were treated with glycine or L-alanine (amino acid control) for up to 15 weeks and voluntary running distance (a quality of life marker and strong correlate of lifespan in *dko* mice) and muscle morphology were assessed. Glycine increased voluntary running distance in *mdx* mice by 90% (P < 0.05) after 2 weeks and by 60% (P < 0.01) in *dko* mice co-treated with prednisolone over an 8 week treatment period. Glycine treatment attenuated fibrotic deposition in the diaphragm by 28% (P < 0.05) after 10 weeks in *mdx* mice and by 22% (P < 0.02) after 14 weeks in *dko* mice. Glycine treatment augmented the prednisolone-induced reduction in fibrosis in diaphragm muscles of *dko* mice (23%, P < 0.05) after 8 weeks. Our findings provide strong evidence that glycine supplementation may be a safe, simple and effective adjuvant for improving the efficacy of prednisolone treatment and improving the quality of life for DMD patients.

## Introduction

Duchenne muscular dystrophy (DMD) is an X-linked neuromuscular disease, which affects around 1:3500-6000 live male births worldwide^1^ and characterized by progressive muscle wasting and weakness. Affected boys usually become wheelchair-dependent by their early teens^2,3^. All muscles are eventually affected including the diaphragm, the primary muscle for breathing. Patients experience a severely reduced quality of life and die prematurely. DMD is caused by a variety of mutations in the dystrophin gene resulting in very low levels or the complete absence of the dystrophin protein, which normally links the actin cytoskeleton to laminin in the extracellular matrix through the dystrophin associated protein complex (DAPC), a key structural element of muscle fibers^3^. Although a cure for DMD may eventually come from corrective gene therapy, limitations in vector carrying capacity, dissemination efficiency, expression persistence, and immunological tolerance, pose significant challenges for clinical application. Until these techniques are perfected, other treatments are needed urgently to counteract the progressive muscle loss and weakness.

Inflammation plays a central role in dystrophic pathology progression. Depletion of inflammatory cells such as eosinophils^4^, T cells^5^, and macrophages^6^ or key mediators of inflammation, such as NF-κB^7^, TNFα^8^, and Toll-like receptor 4 (TLR4)^9^ all ameliorate the dystrophic pathology in *mdx* mice. Muscle fragility and chronic low-grade inflammation increase muscle breakdown and impair regenerative capacity, and as the disease progresses, functional muscle fibers are steadily lost and replaced with fat and connective tissue (i.e. fibrosis). Therefore, interventions that reduce inflammation and modulate the immune response are likely to be therapeutic for DMD patients. To date, glucocorticoids like prednisolone are the only drugs with significant favorable impact on the quality of life of DMD patients. Prednisolone exerts anti-inflammatory^10^ and immunosuppressive^11^ effects on dystrophic muscles, helping to attenuate elevations in [Ca^2+^]_i_ that trigger muscle fiber degeneration^12^, and preserve muscle strength^13^, and functional capacity^14^. Although glucocorticoids are currently the ‘gold standard’ treatment for DMD patients^14^, these drugs have significant side effects, including weight gain (body fat redistribution and fluid retention), high blood pressure, ulcers, growth inhibition (short stature) and osteoporosis due to loss of trabecular bone which increases the risk of spontaneous fractures^15,16^. As such, there is a profound need for adjuvant therapies able to ameliorate the dystrophic condition and improve patient quality of life.

Glycine is a simple amino acid made up of a single carbon molecule, an amino group and a carboxyl group. Glycine is often considered biologically neutral and has even been used as an isonitrogenous control in amino acid supplementation studies. However, glycine is an anti-inflammatory amino acid which effectively blocks inflammatory cell activation^17^. Increased circulating glycine concentrations activate glycine-gated Cl^−^ channels in inflammatory cells, such as macrophages^18^, and thereby blunt pro-inflammatory cytokine production^19^. Indeed, glycine markedly blunts TNFα production in response to hemorrhagic shock and an LPS challenge^20^. We have also demonstrated that glycine treatment effectively counteracts muscle loss in different wasting models, including cancer cachexia^4^, sepsis^21^ and reduced caloric intake^22^. In C26 tumour bearing mice, glycine blunted muscle inflammation, ROS and macrophage infiltration and halved the loss of muscle mass and strength^23^.

Since glycine effectively reduced inflammation and preserved muscle mass in tumour-bearing mice, we proposed that glycine treatment could ameliorate the dystrophic pathology. To test this hypothesis, mildly dystrophic (*mdx*), and severely dystrophic (dystrophin-utrophin null, *dko*) mice were used. The *mdx* dystrophic mouse has a point mutation in the dystrophin gene resulting in an absence of the dystrophin protein. Despite sharing an identical genetic deficit with DMD patients, the dystrophic muscle phenotype in limb muscles of the *mdx* mouse is relatively mild^24^, which is explained, at least in part, by a compensatory upregulation of the functionally similar protein utrophin^25^. On the other hand, the dystrophin-utrophin null (*dko*) mouse, exhibits severe wasting and weakness (in all muscles), spinal deformities (kyphosis) from an early age, and a truncated lifespan, more phenotypically representative of DMD^26^. We hypothesized that glycine administration would blunt pro-inflammatory signaling and the infiltration of fibrosis in dystrophic muscles and attenuate progression of the dystrophic pathology.

## Results

### Glycine improves muscle function and reduces fibrosis in mdx dystrophic mice

**Figure 1:**
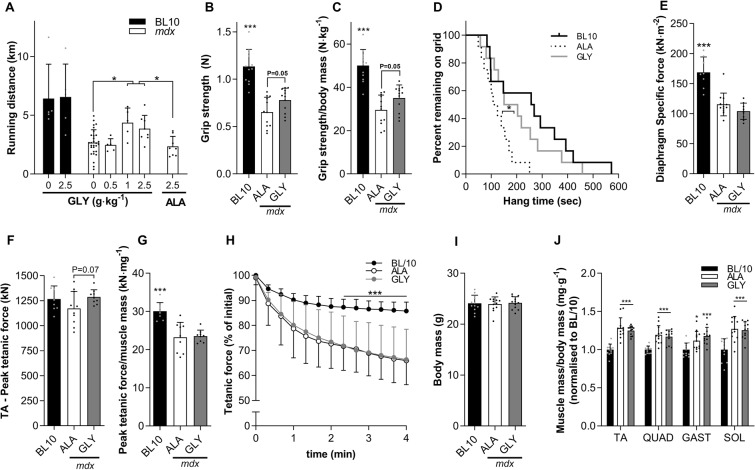
Glycine improves muscle function in mdx dystrophic mice. Dose-response effect of glycine feeding on running performance in *mdx* mice (**A**), with no effect of L-alanine (control). Three weeks of glycine feeding improved whole-body function (**B**–**D**), tended to improve TA peak tetanic force (**F**) but not muscle mass-normalized peak tetanic force (**G**) or diaphragm specific force (**E**), TA fatigue (**H**), body mass (**I**) or muscle mass normalized to body mass and BL/10 (**J**). The data are mean ± SD of 10–12 animals per group. Data were analyzed using one-way ANOVAs with Fishers LSD or Tukey’s (A) post hoc test, except for (D) which was analyzed using a log-rank Mantel-Cox test. *denotes p < 0.05; **denotes p < 0.01; ***denotes p < 0.001.

In an initial dose-ranging study, we used voluntary running distance as a real-time measure of treatment efficacy. Voluntary running distance was 70–90% greater (*P* < 0.05) in *mdx* mice supplemented for 2 weeks with 1 and 2.5 g.kg^−1^.day^−1^ of glycine (i.e. a glycine concentration of 0.75 and 1.9% w/w in food, respectively) compared to a control diet (0% supplemental glycine) (Fig. 1A). To put these changes in perspective, glycine supplementation recovered approximately half the difference in running distance between control BL/10 and dystrophic *mdx* mice. Importantly, an equivalent dose of L-alanine (2.5 g·kg^−1^·day^−1^) did not alter voluntary running distance. In BL/10 mice glycine did not alter voluntary running distance, which was ~180% higher than in *mdx* mice. Based on these results, a dose of 2.5 g·kg^−1^·day^−1^ glycine was used for all further experiments and compared with a control group receiving 2.5 g·kg^−1^·day^−1^ L-alanine.

In a follow-up, short-term (3 week) supplementation study we observed improved whole-body muscle strength in glycine-treated compared with L-alanine-treated *mdx* mice, as evidenced by increased absolute (+20%, *P* = 0.05; Fig. 1B) and body mass-normalized (+19%, *P* = 0.05; Fig. 1C) forelimb grip strength and a longer latency-to-fall on the inverted hang test (+65%, *P* < 0.05; Fig. 1D). *In situ* peak tetanic force, also tended to be higher in glycine- treated, compared with L-alanine-treated *mdx* mice (+10%, P = 0.07, Fig. 1E). Relative TA peak tetanic force, fatigability, muscle mass or diaphragm specific force were not altered by 3 weeks of glycine treatment (Fig. 1F–J).

**Figure 2:**
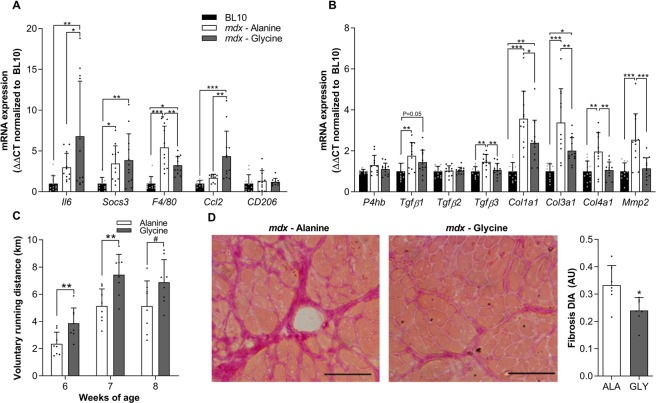
Long-term glycine feeding improves voluntary running and reduces fibrosis in mdx dystrophic mice. The effect of glycine or L-alanine feeding in *mdx* mice on mRNA expression of inflammatory mediators (**A**, interleukin-6, *Il-6*; suppressor of cytokine signaling 3, *Socs3*; EGF-like module-containing mucin-like hormone receptor-like 1, *F4/80*; chemokine ligand 2, *Ccl2*; mannose receptor, *Cd206*) and factors involved in fibrosis/collagen synthesis (**B)**, protein disulphide-isomerase, *P4hb*; transforming growth factor beta, *Tgfβ*; collagen type I, III and IV, *Col1a1*, *Col3a1* and *Col4a1* and; matrix metallopeptidase 2, *Mmp2*). The PCR data are normalized to the BL/10 controls and presented as mean ± SD of 10–12 animals per group. The effect of chronic glycine feeding on running performance in *mdx* mice (**C**) compared to L-alanine (control). Representative muscle cross-sections stained with Van Gieson’s to visualize collagen and its quantification from *mdx* mice after chronic treatment with glycine (**D**). Scale bar represents 100 µm. Data were analyzed using one-way ANOVAs with Fishers LSD post hoc test. *denotes p < 0.05; ** denotes p < 0.01; *** denotes p < 0.001.

Short-term glycine treatment in *mdx* mice suppressed the mRNA expression of selected markers of macrophage infiltration (F4/80: −40%, *P* < 0.05) and collagen turnover (*Col3a1*: −41%, *P* < 0.05; *Col4a1:* −40%, *P* < 0.05 and; *Mmp2*: −55%, *P* < 0.01), while pro-regenerative cytokine and chemokine mRNA expression (*Il6*: +127%, *P* < 0.05 and; *Ccl*2 +158%, *P* < 0.05) was augmented compared to diaphragm muscles from alanine-treated *mdx* controls (Fig. 2A,B). Based on the observed improvements in muscle function and pathological inflammatory and fibrotic signaling, we then hypothesized that longer-term (10 weeks) treatment would reduce fibrotic infiltration in the diaphragm of *mdx* mice. Once again, glycine acutely improved voluntary running distance across the first few weeks of treatment compared to alanine treated controls (Fig. 2C). Furthermore, 10 weeks of glycine treatment led to a significant reduction (−28%, *P* < 0.05) in collagen infiltration in the diaphragm muscle of *mdx* mice (Fig. 2D). The improvements in the pathology were not associated with changes in gross limb or diaphragm muscle fibre size, since no differences were observed between L-alanine and glycine groups for diaphragm muscle fiber cross-sectional area or hind limb (quadriceps and tibialis anterior) muscle mass. Combined, these observations indicate that glycine may attenuate progression of aspects of the dystrophic pathology.

### Glycine blunts diaphragm fibrosis but is insufficient as a standalone treatment to extend lifespan in severely dystrophic dko mice

**Figure 3:**
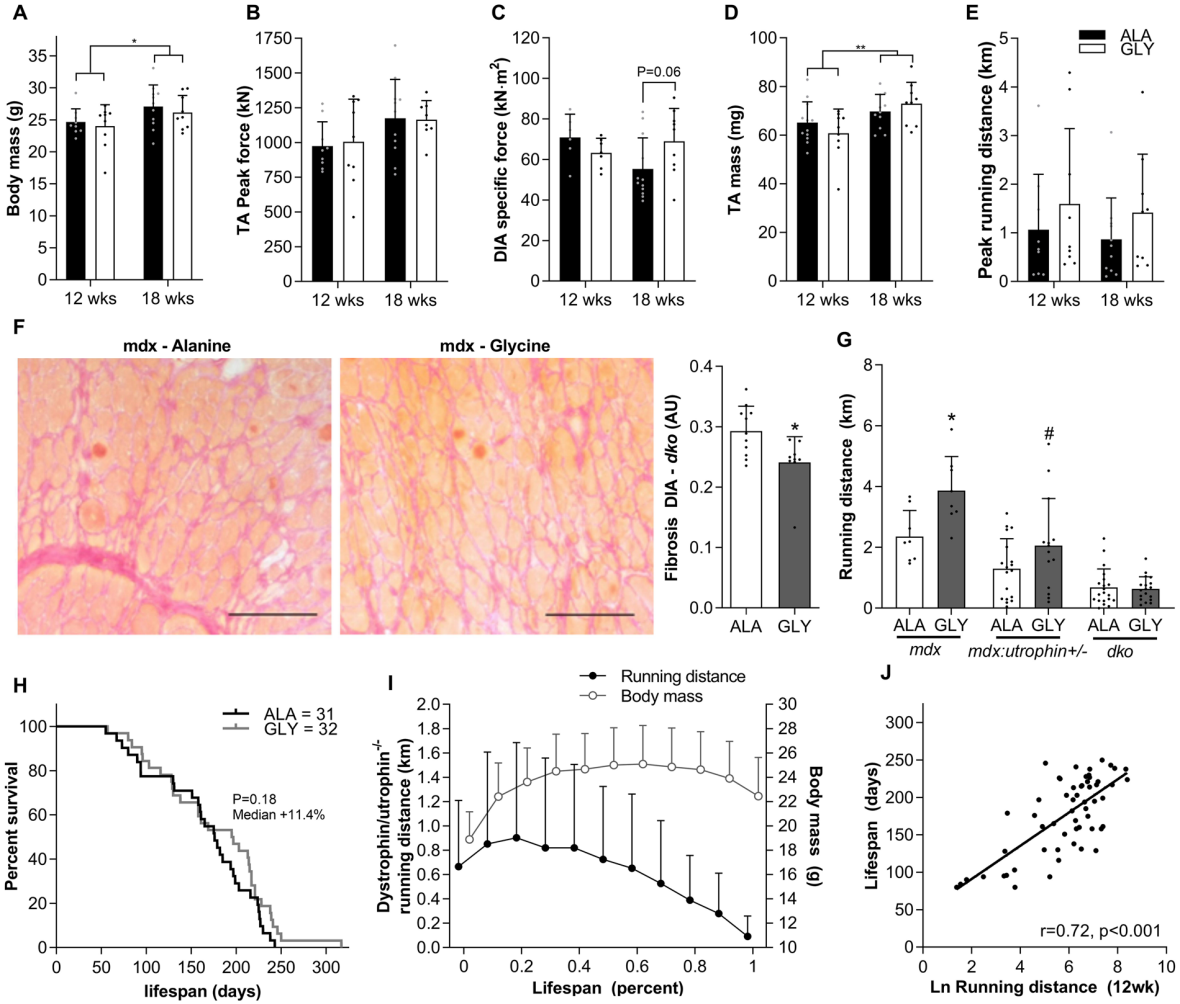
Glycine blunts diaphragm fibrosis but is insufficient as a standalone treatment to extend lifespan in severely dystrophic dko mice. The effect of glycine or L-alanine feeding in *dko* mice on body mass (**A**), TA (**B**) and diaphragm (**C**) muscle function, TA mass (**D**) and voluntary running distance (**E**). Representative muscle cross-sections stained with Van Gieson’s to visualize collagen and its quantification from *dko* mice after chronic treatment with glycine (**F**). Scale bar represents 100 µm. Voluntary running distance in *mdx*, *mdx:utrophin*^+/−^ and *dko* treated with either glycine or L-alanine for 2 weeks (**G**). Survival curve of L-alanine and glycine-treated *dko* mice (**H**); changes in running distance and body mass throughout the lifespan of *dko* mice (**I**); and correlation (Pearson’s r) between lifespan and the natural logarithm of running distance (**J**). Data in A-E were analyzed using two-way ANOVAs with Sidak’s post hoc test. Two-group comparisons were made using students t-tests, which lifespan curves were compared using a log-rank Mantel-Cox test. Data are mean ± SD of at least 8 mice per group.

Since the dystrophic phenotype of *mdx* mice is comparatively mild to that of human *DMD* patients we tested the efficacy of glycine treatment in severely dystrophic dystrophin-utrophin double knockout (*dko*) mice. To test whether glycine could attenuate the progression of the pathology in these more severely dystrophic mice, we treated 4-week-old *dko* mice for 8 or 14 weeks with either glycine or L-alanine (Fig. 3A–F). Glycine treatment tended to increase diaphragm specific force in 14-week treated mice (+25%; *P* = 0.06, Fig. 3C), but not in 8-week treated mice. Glycine did not increase peak tetanic force in the TA muscle (Fig. 3B) nor peak voluntary running distance in *dko* mice (Fig. 3E). However, in line with experiments in *mdx* mice, diaphragms from glycine treated mice had significantly less collagen infiltration (−18%, *P* < 0.05) than L-alanine-treated controls (Fig. 3F). To determine whether disease severity affected the efficacy of glycine treatment, we examined voluntary running capacity in *mdx* mice with heterozygous utrophin expression after 2 weeks of glycine treatment and compared the result of voluntary running distance after a similar treatment period in *mdx* and *dko* mice. Glycine treatment increased voluntary running distance by + 1507 m or 64% (*P* < 0.05) over a 24 h period while glycine only tended to improve voluntary running distance (761 m, 59%, *P* = 0.10) in *mdx utrophin*^+/−^ mice and did not improve running distance in *dko* mice (Fig. 3G). Glycine treatment was not sufficient to improve the lifespan of severely dystrophic *dko* mice (Fig. 3H). Combined, these findings suggest that glycine as a standalone treatment may attenuate progression of the dystrophic pathology, although its efficacy may be limited in those most severely affected.

### Voluntary running distance is strongly associated with disease severity and predicts lifespan in severely dystrophic dko mice

We identified voluntary running distance as a sensitive and specific measure of disease severity in dystrophic mice. Running distance over a 24 h period progressively decreased from BL/10 to *mdx* to *mdx*:utrophin^+/−^ to *dko* mice (Fig. 3G). Furthermore, we recorded 24 h voluntary running distance along with body mass and kyphosis index on a weekly basis across the entire lifespan of glycine and L-alanine treated *dko* mice starting from 4-weeks of age. Running capacity progressively decreased beyond 50% of lifespan in *dko* mice and was ~90% lower than peak levels in the week prior to reaching humane endpoint criteria. In contrast, body mass was only reduced in the final 20% of lifespan and by ~15% in the week prior to endpoint (Fig. 3I). While body mass and kyphosis between 8–20 weeks correlate with lifespan, running distance most strongly correlated with lifespan (Table 1). Multiple regression analysis using the two objective measures of body mass and running distance yielded stronger relationships than the subjective measure of kyphosis at each time point (Table 1). Including kyphosis in the model slightly improved the predictive relationship at weeks 8, 10 and 14, but not at any other time point. Voluntary running distance assessed at 12 weeks of age strongly predicted lifespan (Fig. 3J), explaining approximately 50% of the variation in lifespan. These findings demonstrate that voluntary running distance is as an objective, simple and real-time measure of pathological progression and treatment efficacy in dystrophic mice.

**Table 1:** Correlates of dko lifespan.

Age (wks)	Kyphosis	Ln RD	BM (g)	BM and Ln RD	BM, Ln RD and KI
8	−0.55***	0.49***	0.42**	0.57***	0.61***
10	−0.53***	0.57***	0.50***	0.63***	0.68***
12	−0.58***	0.75***	0.58***	0.78***	
14	−0.62***	0.67***	0.58***	0.75***	0.77***
16	−0.58***	0.60***	0.54***	0.67***	
18	−0.54***	0.73***	0.60***	0.79***	
20	−0.48**	0.57***	0.55***	0.66***	

Correlations (pearsons r) between lifespan and kyphosis index (KI), the natural logarithm of running distance (Ln RD), body mass (BM), and multiple regression correlation for lifespan using body mass and Ln RD only or all three measures. Only statistically significant (*P* < 0.05) correlations are reported. Data are reported for measures taken every two weeks between 8 and 20 weeks of age. ** and *** denote significant correlations at the *P* < 0.01 and *P* < 0.001 level.

### Glycine treatment augments prednisolone-induced improvements in voluntary wheel running and diaphragm fibrosis in dko mice

**Figure 4:**
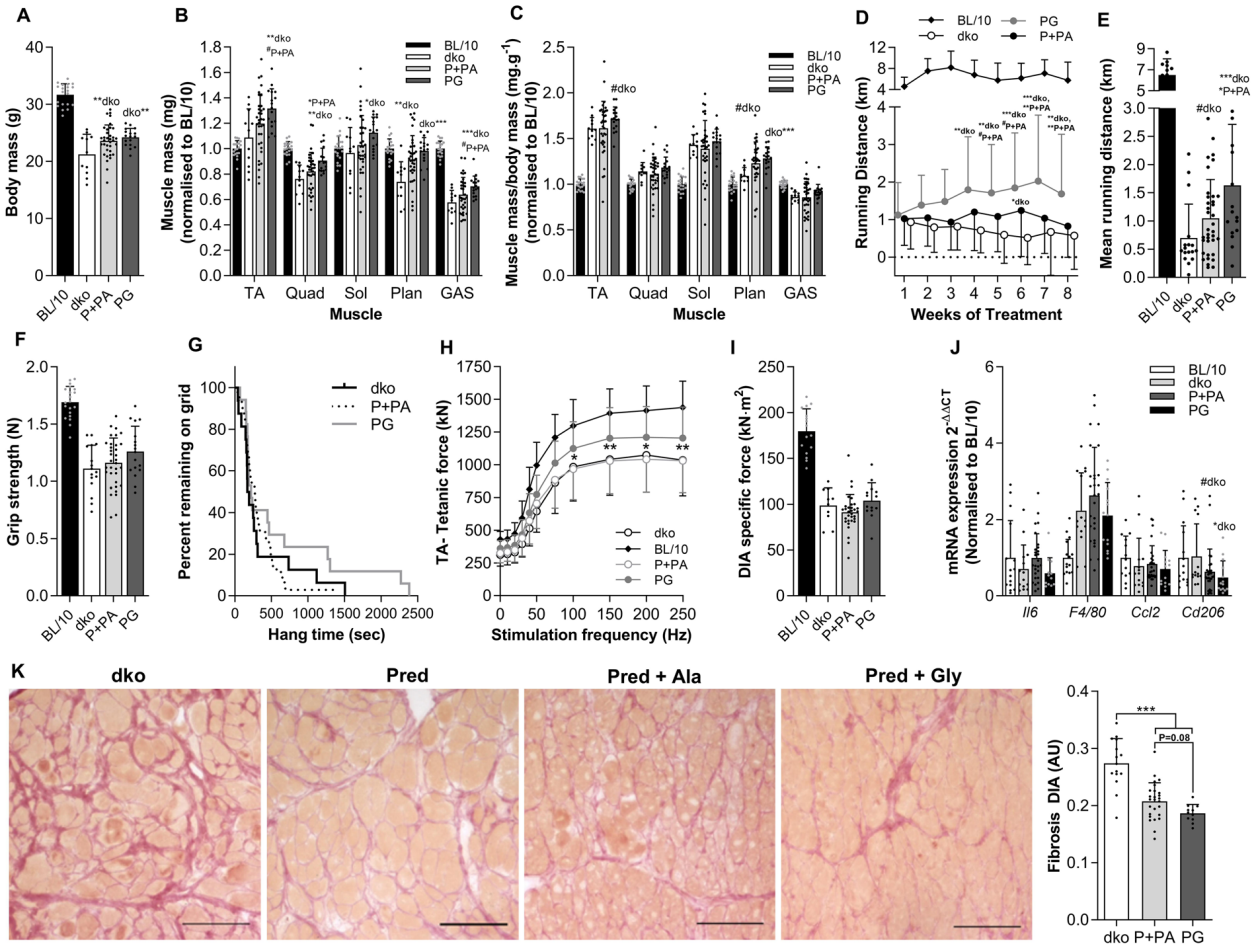
Glycine treatment augments prednisolone-induced improvements in voluntary wheel running and diaphragm fibrosis in dko mice. Endpoint body mass (**A**), absolute muscle mass normalized to BL/10 (**B**) muscle mass normalized to body mass and BL/10 (**C**), as well as weekly (**D**) and average (**E**) voluntary running distance in *dko* mice receiving prednisolone with either glycine or L-alanine for 8 weeks. Individual muscle mass in *dko* mice after 8 weeks of treatment (**C**). Assessments of whole body function including forelimb grip strength (**F**) and inverted hang time (**G**). The effect of glycine and prednisolone treatment on *in situ* peak tetanic force of the TA muscle at different stimulation frequencies (**H**), *ex vivo* peak specific force of diaphragm muscle in *dko* mice (**I**) and inflammatory gene expression (**J**). Representative muscle cross-sections stained with Van Gieson’s to visualize collagen and its quantification from *dko* (**K**) mice after 8 weeks of prednisolone and glycine treatment. Scale bar represents 100 µm. Data were analyzed using one-way ANOVAs with Fishers LSD post hoc test. * denotes p < 0.05; ** denotes p < 0.01; *** denotes p < 0.001. Data are presented as mean ± SD for 12–15 mice per group.

Since glycine effectively improved pathological features in mildly dystrophic *mdx* mice but was not sufficient as a standalone treatment in more severely dystrophic *dko* mice, we hypothesized that glycine could improve the dystrophic phenotype in combination with the current gold-standard *DMD* treatment, prednisolone. To this end, we treated 4-week-old *dko* mice 3 times per week for 8 weeks with 5 mg·kg^−1^ prednisolone or a vehicle orally. In addition, mice received either glycine, L-alanine, or non-supplemented food. Since prednisolone-treated mice receiving L-alanine and non-supplemented food recorded identical results and the inter-mouse variability of *dko* mice is large, we pooled these two groups for analysis. Mice had access to a running wheel for 24 h once a week for the 8-week treatment period to assess voluntary running distance as a real-time proxy for disease progression. Throughout the 8-week treatment period, running distance tended to be greater in prednisolone treated *dko* mice compared with vehicle treated *dko* mice (Fig. 4D,E). Co-treatment of *dko* mice with prednisolone and glycine significantly improved voluntary running distance compared to vehicle-treated *dko* and prednisolone-treated *dko* after 4 and 7 weeks of treatment, respectively (Fig. 4D). Mean running distance across the 8-week treatment period was significantly greater in prednisolone- and glycine-treated *dko* mice compared with vehicle- (+133%, *P* < 0.01) or prednisolone-treated (+56%, *P* < 0.05) *dko* mice (Fig. 4E). While prednisolone did not alter absolute muscle mass, except in the plantaris, co-treatment with prednisolone and glycine significantly increased absolute tibialis anterior (TA, +21%; *P* < 0.01), quadriceps (+19%; *P* < 0.01), soleus (16%; *P* < 0.05), plantaris (33%; *P* < 0.01) and gastrocnemius (22%; *P* < 0.001) muscle mass compared to vehicle treated *dko* mice (Fig. 4B). Glycine also increased plantaris and tended to increase TA muscle mass normalized to body mass (Fig. 4C). Glycine tended to increase absolute TA (+10%; *P* = 0.06), quadriceps (+10%; *P* < 0.05) and gastrocnemius (+10%; *P* = 0.07) muscle mass compared to prednisolone treated *dko* mice (Fig. 4B). The improvement in hindlimb muscle mass also translated into significantly higher *in situ* peak tetanic force in the TA muscles (Fig. 4H), but no improvements in forelimb grip strength or inverted hang time (Fig. 4F,G). The improvements in skeletal muscle mass occured in the absence of changes in body mass between prednisolone and prednisolone and glycine treated mice (Fig. 4A). Inflammatory signaling was not altered in *dko* mice after the 8 week treatment period, but prednisolone and glycine treated mice had lower levels of *CD206* mRNA compared to *dko* controls (Fig. 4J). Eight weeks of prednisolone treatment significantly reduced collagen infiltration (−24%; *P* < 0.0001) in the diaphragm muscle of *dko* mice (Fig. 4K). Glycine treatment tended to further reduce diaphragm collagen infiltration compared to prednisolone treated (−10%; *P* = 0.08) *dko* mice such that collagen infiltration was 32% less (P < 0.0001) than in vehicle treated *dko* mice.

## Discussion

Glycine administration effectively reduces [Ca^2+^]_i_ and cytokine production in inflammatory cells via the activation of glycine-gated chloride channels, thereby reducing whole-body (systemic) inflammation^27^. Since DMD is characterized by chronic low-grade inflammation^28^, glycine supplementation could represent an effective treatment. We showed that incorporation of glycine (1.9% w/w) into a semi-purified chow diet acutely improved voluntary running distance in *mdx* mice and prolonged glycine treatment (10–15 weeks) reduced fibrosis in the diaphragm of *mdx* and *dko* dystrophic mice. To determine the clinical potential for glycine to attenuate progression of the dystrophic pathology, we also assessed the efficacy of glycine feeding in mice treated simultaneously with prednisolone, the current gold-standard treatment for DMD. Combined prednisolone and glycine treatment improved voluntary running capacity, a strong predictor of disease severity and lifespan in *dko* mice, and attenuated deposition of fibrosis in the diaphragm. Overall, our findings highlight the therapeutic potential of glycine for improving clinically-relevant functional measures and slowing the pathologic progression in DMD.

### Voluntary running distance is a real-time indicator of disease severity

In young DMD patients, walking tests provide a critical measure of physical function and mobility and are used to assess pathological progression and efficacy of interventions^29,30^. Similarly, measures of activity or running ability are commonly used as reliable and sensitive quality-of-life measures in dystrophic mouse models^31–35^, with *mdx* and *dko* mice exhibiting a marked reduction in treadmill and wheel running capacity compared with BL/10 mice. Although the disease severity and lifespan varies widely between *dko* mice (55–246 days in the current study), they generally exhibit a severe pathology and reduced lifespan^26^. We have identified voluntary running distance as a strong predictor of disease severity in mouse models of *DMD*. Voluntary running distance progressively decreases from BL/10 wild-type mice to mildly affected *mdx* mice, moderately affected *mdx*;utrophin^+/−^ mice and severely affected *dko* mice (Fig. 3G). Voluntary running distance also progressively declines throughout the lifespan of *dko* mice, reaching less than 10% of peak values within the last week of life (Fig. 3I). In fact, *dko* lifespan strongly correlates with either peak running distance or running distance at a defined age, e.g. at 12 weeks. Furthermore, using multiple regression analyses, we determined that combined objective measures of body mass and running distance achieved a stronger correlation with lifespan than the more subjective measure of kyphosis index. These data provide strong supporting evidence that measures of running distance provide an accurate assessment of pathological progression in dystrophic mice.

### Glycine improves voluntary running distance and reduces fibrosis in the diaphragm of dystrophic mdx and dko mice

In mice, voluntary wheel running capacity is reduced during inflammatory episodes and restored by anti-inflammatory medication and analgesics^31^. Inflammation plays a critical role in the progression of the dystrophic pathology^4–9^. Glycine can directly modulate inflammation by activating glycine-gated chloride (Cl^−^) channels (GlyR) expressed in inflammatory cells, such as macrophages, and thereby blunting pro-inflammatory cytokine production^36^. Importantly, glycine does not impact the muscle cell’s inflammatory response^21^ which is necessary for successful muscle fibre regeneration^37^. We have previously demonstrated that glycine reduces muscle inflammation, ROS and macrophage infiltration in tumour-bearing mice, thereby halving the loss of muscle mass and strength^23^. Therefore, in the present study, we assessed whether glycine treatment could improve voluntary running distance in treated *mdx* mice and whether glycine treatment changed the inflammatory signature in skeletal muscle. In 6-week-old *mdx* mice, we observed a ~65% reduction in voluntary wheel running distance over a 24 h period compared to age-matched BL/10 controls (Fig. 1A). Compared to 6-week-old *mdx* mice fed a standard diet, 2–3 weeks of glycine treatment improved whole-body (voluntary running distance, grip strength, latency-to-fall) and *in situ* TA muscle function in *mdx* mice (Fig. 1). Short-term glycine treatment in *mdx* mice reduced the mRNA expression of F4/80 suggesting a reduction in macrophage infiltration but increased mRNA expression of *Il6* and *Ccl2* (Fig. 2A). While IL6 and CCL2 are traditionally considered pro-inflammatory, skeletal muscle tissue expression of both IL6^38^ and CCL2^39^ play crucial roles in muscle regeneration. Notably, the *mdx* mice used in this experiment were examined at an age associated with high macrophage infiltration and frequent cycles of degeneration/regeneration^6^. Furthermore, glycine reduced mRNA expression of factors related to fibrosis, such as *Tgfb1*, *Tgfb3*, *Col3a1*, *Col4a1* and *Mmp2* (Fig. 2B). Together, these data support the hypothesis that glycine treatment can improve skeletal muscle performance in *mdx* dystrophic mice and led us to perform long-term studies investigating whether glycine treatment could slow fibrotic deposition in dystrophic muscles in two murine models of DMD.

In line with the observed suppression of fibrotic markers following short-term glycine treatment, 10 weeks of glycine treatment reduced fibrotic infiltration by 28% in the diaphragm muscles of *mdx* mice (Fig. 2D). In *mdx* mice, the diaphragm exhibits a more severe and progressive dystrophic pathology than limb muscles with marked fibrotic infiltration and is comparable to that observed in DMD^24^. While the 30% reduction in fibrosis in the diaphragm with glycine treatment represents only an ~5% improvement in functional muscle fibers, which may not translate to measurable improvements in isometric muscle force, the reduction in fibrosis is comparable to that observed after chronic administration with anti-fibrotic agents, such as Tranilast^40^. It is also possible that compensatory mechanisms, such as GlyR desensitization^41^, blunts the beneficial effects of glycine treatment in long-term treatment studies. Together, the marked improvement in voluntary running distance in glycine treated *mdx* mice, combined with the reduction in fibrosis after 10 weeks of treatment in *mdx* mice supports our hypothesis that glycine has therapeutic potential for DMD.

### Glycine is insufficient as a standalone treatment to extend lifespan in severely dystrophic dko mice

Glycine’s effect on running distance was less pronounced in *mdx*:utophin^+/−^ mice and absent in *dko* mice, suggesting a relationship between treatment efficacy and disease severity and/or stage of disease progression. Indeed, despite improvements in diaphragm fibrosis and a tendency for improved diaphragm specific force at 18 weeks of age in glycine treated *dko* mice, glycine did not improve peak *in situ* force in the TA muscle and was incapable of extending lifespan. These observations further support the concept of a relatively narrow therapeutic window in which glycine could realistically improve the dystrophic pathology^42^.

### Prednisolone treatment improves voluntary wheel running and reduces fibrosis in the diaphragm of severely dystrophic dko mice

We demonstrated that voluntary running distance tracks with pathological progression from an early age in *dko* mice and this is the first study to show that prednisolone increased voluntary running in these severely dystrophic mice. Previous studies using prednisolone in *mdx* mice have produced variable results which are seemingly dependent on dose and frequency of administration. The effects of prednisolone in *mdx* mice range from no changes^43^, to only moderate effects in limb skeletal muscles^44^ and diaphragm^45,46^, or dramatic increases in strength and function^47^. Our observations are consistent with those of Morrison-Nozik *et al*.^48^, who showed prednisolone treatment increased running capacity in *mdx* mice by 50% using the same dosing regimen. Although we did not observe differences in skeletal muscle mass, fat mass or heart mass between prednisolone and vehicle treated *dko* mice, the reduction in fibrosis in the diaphragm muscle was comparable to that observed with glycine administration in *mdx* and *dko* mice.

To test the clinical potential for glycine therapy to attenuate progression of the dystrophic pathology, we assessed treatment efficacy in 4-week-old *dko* mice also receiving prednisolone (5 mg/kg, 3 times a week for 8 weeks). Running distance was greater in prednisolone treated *dko* mice receiving glycine than L-alanine, providing evidence for a sustained attenuation of the disease progression in glycine-treated mice. Although we did not observe differences in whole-body muscle strength/mobility in glycine-treated compared with alanine-treated *dko* mice receiving prednisolone, quadriceps, gastrocnemius and TA muscle masses were significantly heavier in glycine-treated than alanine-treated control mice. This attenuated muscle wasting or enhanced growth with glycine administration is consistent with our observations in animal models of cancer cachexia^23^ and calorie restriction^22^, where glycine treatment attenuated muscle wasting by up to 50%. Despite the differences in quadriceps, gastrocnemius and TA muscle mass, there was no difference in body mass in *dko* mice, suggesting the sparing of muscle mass was counterbalanced by a reduction in fat mass in glycine treated mice. Similar effects have been observed with glycine feeding during calorie restriction, where glycine enhanced fat loss while preserving skeletal muscle mass^22^.

While the current dose of 1–2.5 g.kg.day^−1^ glycine in mice may seem difficult to achieve in patients, it is important to consider that mice have a higher metabolic rate and food consumption, such that protein intake in mice is ~25 fold higher than in humans^23^. Assuming the dose of glycine required to exert effects in humans is similarly scaled, the glycine dose of 1–2.5 g·kg^−1^·day^−1^ in mice would equate to a highly achievable daily glycine intake of 3–7 g for a 70 kg human. Further studies would be required to establish an effective dose and ingestion regimen in humans.

Glycine is a simple, safe and inexpensive non-essential amino acid with well-described effects on inflammatory cells^36^. Here we identify a novel therapeutic use for glycine in the treatment of muscular dystrophy. Glycine blunts fibrotic infiltration and improves whole-body, clinically relevant, measures of muscle function in *mdx* and prednisolone-treated *dko* mice. Together, these data highlight the suitability of glycine as an adjunct nutritional intervention for the treatment of muscular dystrophies.

## Conclusion

These comprehensive data from two murine models of DMD provide strong evidence that glycine can attenuate the progression of the dystrophic pathology. Moreover, glycine supplementation can improve the efficacy of prednisolone, the current gold-standard treatment for DMD patients, in severely dystrophic mice. Taken together with its established safety and efficacy, our findings highlight glycine’s therapeutic potential in DMD and strongly support clinical trials.

## Methods

### Animals

All experiments were conducted in accordance with the Australian code of practice for the care and use of animals for scientific purposes as outlined by the National Health and Medical Research Council of Australia (Canberra, ACT, Australia) and approved by the Animal Ethics Committee of The University of Melbourne. Four-week-old male C57BL10, BL/10ScSn-*mdx*/J (*mdx)*, and dystrophin/utrophin ‘double knockout’ (*dko*) mice^25^ with a more severe dystrophic pathology were used in this study. *Mdx* mice were obtained from the Animal Resources Centre (ARC, Canning Vale, Western Australia), while *dko* mice were bred as described previously^25,26,42^. Briefly, female *mdx* breeders (sourced from the ARC) were bred with male utrophin knockout mice (Utrn^−/−^). F1 generation females were then mated with Utrn^−/−^ males, with an expected male dystrophin/utrophin-null or *dko* yield of 25%. All animals were housed in the Biological Research Facility at The University of Melbourne under a 12:12-hour light-dark cycle and monitored daily for adverse signs and symptoms.

### Experimental outline

For the initial dose-response experiments, four-week-old *mdx* mice were fed a purified diet containing either 0, 0.375, 0.75 or 1.9% glycine, equating to 0, 0.5, 1 and 2.5 g·kg^−1^·day^−1^ glycine (n = 5 per group), for two weeks and then housed individually for 24 h with access to a running wheel. In addition, four-week-old BL/10 (n = 12) or *mdx:utrophin*^+/−^ (n = 15) mice were fed a diet containing either 0% or 1.9% glycine (2.5 g·kg^−1^·day^−1^) for two weeks and then housed individually for 24 h with free access to a running wheel. Following the running wheel assessments, whole-body mobility and strength were evaluated and mice were killed after three weeks of treatment. For the prolonged supplementation studies, four-week-old male *mdx* mice (n = 8 per treatment group) and *dko* mice (n > 9 per treatment group) were fed a purified diet containing 1.9% glycine (GLY) or 1.9% L-alanine (ALA) for 10 (*mdx*) and 8 or 14 (*dko*) weeks, respectively.

For *dko* lifespan experiments, male mice were fed 1.9% L-alanine (n = 31) or 1.9% glycine (n = 32) until they reached a humane endpoint. The criteria used to determine when a mouse had reached a humane endpoint were: (1) a loss of >15% body mass; (2) not eating or drinking for >24 h; or (3) a body condition score of 2- or less. Confirmation that a humane endpoint had been reached was validated by an experienced independent animal house staff member for each mouse. Body mass and kyphosis index were recorded three times per week and mice were housed individually with free access to a running wheel once per week for their entire lifespan.

To assess whether glycine and prednisolone can slow disease progression in severely dystrophic mice, four-week-old male *dko* mice (n = 17–18 per treatment group) were allocated into the following treatment groups: (1) *dko* + vehicle; (2) *dko* + prednisolone; (3) *dko* + pred + L-alanine and; (4) *dko* + pred + glycine. Prednisolone (5 mg/kg) or vehicle (fruit syrup) were administered three times per week via oral gavage while glycine and L-alanine were administered at 1.9% w/w via the food, as in previous experiments. Once per week mice were housed individually in boxes with running wheel access for 24 hours. Whole-body functional assessments were performed prior to sacrifice. For all experiments, mice were killed *via* cardiac excision while under deep anesthesia and selected tissues were collected as described^22,23^. Researchers were not blinded to treatment groups except during assessments of muscle function.

### Amino acid administration

L-alanine and glycine (Sigma-Aldrich Co., Castle Hill, NSW; Australia) were incorporated into a color0-coded semi-pure growth diet (AIN93G;^22^ Specialty Feeds, WA, Australia). Based on our previous experiments^21–23^, we used the non-essential amino acid L-alanine as an isonitrogenous control to ensure that any differences observed were not simply the result of an increased nitrogen intake. Based on initial dose-response experiments (Fig. 1), a glycine dose of ~2.5 g·kg^−1^·day^−1^ or a chow concentration of 1.9% w/w glycine was used. No difference in food consumption was observed between GLY and ALA groups in *mdx* or *dko* mice. The protein content of the AIN93G diet was ~20%, and therefore, the glycine dose administered represents a modest increase in nitrogen intake of <10%.

### Prednisolone administration

Prednisolone (5 mg/kg body weight) was administered three times per week, as described previously^48^. Briefly, prednisolone (40 mg/ml, Sigma-Aldrich Co., Castle Hill, NSW, Australia) was dissolved in ethanol/PBS/fruit syrup (70:20:10) solutions. This stock solution was further diluted in fruit syrup to achieve a prednisolone concentration of 2.86 mg/ml. This prednisolone syrup was administered *via* oral gavage (1.75 uL/g body weight). No adverse reactions were observed following administration.

### Voluntary running

Based on the natural propensity of mice to run, spontaneous wheel running activity over a 24 h period is commonly used as a reliable and sensitive quality-of-life index^31–33^. Mice were housed individually with running wheel access (Activity wheel, model 80820, Lafayette instrument, Indiana, USA) for a 24 h period once per week.

### Whole body muscle function

Whole body mobility and coordination was assessed by rotarod performance (Rotamex-5, Columbus Instruments)^49^. The test was performed as described previously^23^. Briefly, mice were placed onto a rod rotating at 4 rpm. Speed increased 1 rpm every 8 sec until mice fell onto a soft pad. The latency-to-fall was recorded as the duration on the rod before falling across three trials, with 15 min rest between trials.

Whole body strength was assessed with a grip strength meter (Columbus Instruments, Columbus, OH)^49^. The test was performed as described previously^23^. Briefly, Mice were allowed to grab a triangular metal ring connected to a force transducer and were then gently pulled by the tail until the grip was broken. The test was performed five times in a 2 min period and the maximum peak force (N) was recorded. For both assessments, mice were acclimatized to the testing room for 15 min and tests were performed in an alternating order between groups, with one full box of mice tested at a time.

### *In situ* TA muscle function

At the end of treatment, mice were anesthetized with sodium pentobarbitone (Nembutal, 60 mg/kg, Sigma-Aldrich) via *i*.*p*. injection until they were unresponsive to tactile stimuli. An appropriate depth of anaesthesia was maintained via supplementary doses. Contractile properties (maximal force, fatigue and recovery) of the right tibialis anterior (TA) muscle were assessed *in situ*, as described by us in detail elsewhere^50,51^. Briefly, the TA muscle was exposed and the distal tendon securely tied to the lever arm of a dual mode servomotor/force transducer coupled to a force-length controller (dual-mode lever system, 300B-LR; Aurora Scientific Inc, Aurora, Ontario, Canada). Supra-maximal (14 V) 0.2-ms square wave pulses of 350 ms in duration were used to stimulate the TA muscle via the peroneal nerve (a branch of the sciatic nerve). The servomotor-computer interface was controlled by custom-written software (D.R. Stom Inc, Ann Arbor, Michigan, USA) running LabView (LabVIEW 5.1; National Instruments, Austin, Texas, USA). Using a 1 Hz stimulation frequency, muscle length was adjusted carefully until a maximum isometric twitch (P_t_) response was obtained at the optimum muscle length (L_o_), which was measured with electronic digital calipers. Maximum isometric tetanic force (P_o_) was taken as the plateau of the frequency-force relationship, with the muscle being stimulated at 1, 10, 20, 30, 40, 50, 75, 100, 150, 200, 250, and 300 Hz. After each tetanic contraction the muscle was rested for 2 min to prevent fatigue. Specific muscle force (kN·m2), representing peak force normalised to cross-sectional area, was calculated using the formula: peak force (mN)/[muscle mass (g)/0.636·muscle length (mm)], where 0.636·muscle length is used to estimate cross-sectional area, as described in detail elsewhere^51^.

### *In situ* diaphragm function

Contractile properties of diaphragm muscle strips were assessed *ex vivo*, as described previously^52^. Briefly, the diaphragm and rib cage were surgically excised and costal diaphragm muscle strips composed of longitudinal, full-length muscle fibers were isolated and prepared for functional assessment *ex vivo*. On completion of functional testing, diaphragm strips were trimmed of tendon and non-muscle tissue, blotted on filter paper and weighed before being mounted in embedding medium and frozen in thawing isopentane for later histochemical analyses.

### Skeletal muscle histology

Serial sections (5 μm) were cut transversely through the right TA muscle of *mdx* mice from GLY and ALA groups using a refrigerated (−20 °C) cryostat (CTI Cryostat; IEC, Needham Heights, MA). Sections were stained with hematoxylin and eosin (H&E) for visualization of general muscle architecture^26^. Muscle collagen content was assessed from Van Gieson-stained cross-sections as described previously^26^. Digital images of stained sections were captured using an upright microscope with camera (Axio Imager D1, Carl Zeiss, Wrek Göttingen, Germany), controlled by AxioVision AC software (AxioVision AC Rel. 4.8.2, Carl Zeiss Imaging Solutions, Wrek, Wrek Göttingen, Germany). Images were quantified using AxioVision 4.8.2 software as described previously.

### RNA extraction and qPCR

Total RNA was extracted from 10–20 mg of diaphragm muscle using the RNeasy Mini Kit (QIAGEN, VIC, Australia) as described previously^21^. RNA concentration and purity were examined using the Nanodrop 1000 (Thermo-Fisher Scientific, VIC, Australia). First-strand cDNA was generated using the iScript™ Reverse Transcription Supermix (Bio-Rad Laboratories, NSW, Australia). qPCR was performed in duplicate (Fig. 1) or triplicate (Figs 2 and 3**)** with reaction volumes of 10 μl, containing SsoAdvanced™ Universal SYBR® Green Supermix (Bio-Rad Laboratories), forward and reverse primers and cDNA template (2 ng/μl) using the Bio-Rad CFX384 PCR system (Bio-Rad Laboratories). Data were analysed using a comparative quantification cycle (Cq) method where the amount of target was normalized to the amount of endogenous control (2^−ΔΔCq^). The efficacy of *Rplp1* as an endogenous control was examined using the equation 2^−ΔCq^. As expression did not differ between treatment groups (data not shown), *Rplp1* represents an appropriate endogenous control. Data were further normalized to BL10 (Fig. 1) or *dko* control mice (Figs 2 and 3) for comparative purposes. We designed primers and confirmed specificity using NCBI primer Basic Local Alignment Search Tool (BLAST). To confirm the generation of a single amplified product, we generated a melting point dissociation curve for all PCR products using the PCR instrument. Primer sequences are listed in Table 2 or described elsewhere^21,22,40^.

**Table 2:** Primer sequences^a^.

Gene	Accession no.	Forward (5′-3′)	Reverse (5′-3′)	Amplicon length
*Cd206*	NM_008625	GTGGAGTGATGGAACCCCAG	CTGTCCGCCCAGTATCCATC	120
*P4hb*	NM_011032	AAGCTGCCGCAAAACTGAAG	TCACTTCGCTTGAGTCCACC	273
*Rplp1*	NM_018853	GGCAGTCTACAGCATGGCTT	GAAAGGTTCGACGCTGACAC	139

^a^Primers were designed using NCBI primer BLAST and specificity confirmed using BLAST. *Cd206* is also known as Mannose receptor C type 1 (Mrc1). *Rplp1*, ribosomal protein large P1.

### Statistical analysis

All values are expressed as mean ± SD unless stated otherwise. Data were tested for normality and homogeneity of variance using a Shapiro-Wilk and Levene’s test, respectively. One-way ANOVAs with Fisher’s LSD post-hoc test were used to compare between groups running distance, grip-strength, hang time and gene expression in the short-term experiments using treated *mdx* groups and control BL/10 groups. Unpaired t-tests were used to make two-group comparisons, while Fisher’s LSD post-hoc tests were used to make three-group comparisons only if the ANOVA *P* value was <0.05, a prerequisite of the test which accounts for multiple comparison bias for comparisons between no more than 3 groups. Correlations between two single variables were assessed by Pearson’s correlation coefficient and a two-tailed significance test. Stepwise multiple regression analysis was used to determine which real-time measures (i.e. kyphosis, body mass or Ln-running distance) best contributed to predictions of lifespan. For stepwise multiple regression, the analysis was first performed using body mass and Ln-running distance only and was then performed using all three independent variables. For comparisons of running distance over time in the prednisolone experiment, between-group comparisons were made using a two-way repeated measures ANOVA with Fisher’s LSD post-hoc test. The BL/10 group is included in Fig. 4 for context only and was not included in statistical analyses. Outliers were removed from RT-qPCR data using the outlier labeling rule and a factor of 2.2^53^. Data were normalized to the appropriate control group (BL/10 or untreated *dko*) for ease of visualization, unless stated otherwise. Both significant differences (P < 0.05) and trends (P < 0.1) are reported where appropriate.

## References

[1] Bushby K (2010). Diagnosis and management of Duchenne muscular dystrophy, part 2: implementation of multidisciplinary care. Lancet Neurol.

[2] Emery AE (2002). The muscular dystrophies. Lancet.

[3] Campbell KP (1995). Three muscular dystrophies: loss of cytoskeleton-extracellular matrix linkage. Cell.

[4] Wehling-Henricks M (2008). Major basic protein-1 promotes fibrosis of dystrophic muscle and attenuates the cellular immune response in muscular dystrophy. Hum Mol Genet.

[5] Morrison J, Lu QL, Pastoret C, Partridge T, Bou-Gharios G (2000). T-cell-dependent fibrosis in the mdx dystrophic mouse. Lab Invest.

[6] Wehling M, Spencer MJ, Tidball JG (2001). A nitric oxide synthase transgene ameliorates muscular dystrophy in mdx mice. J Cell Biol.

[7] Acharyya S (2007). Interplay of IKK/NF-kappaB signaling in macrophages and myofibers promotes muscle degeneration in Duchenne muscular dystrophy. J Clin Invest.

[8] Radley HG, Davies MJ, Grounds MD (2008). Reduced muscle necrosis and long-term benefits in dystrophic mdx mice after cV1q (blockade of TNF) treatment. Neuromuscular disorders: NMD.

[9] Giordano Christian, Mojumdar Kamalika, Liang Feng, Lemaire Christian, Li Tong, Richardson John, Divangahi Maziar, Qureshi Salman, Petrof Basil J. (2014). Toll-like receptor 4 ablation in mdx mice reveals innate immunity as a therapeutic target in Duchenne muscular dystrophy. Human Molecular Genetics.

[10] Hudecki MS (1993). Strength and endurance in the therapeutic evaluation of prednisolone-treated MDX mice. Research communications in chemical pathology and pharmacology.

[11] Kissel JT, Burrow KL, Rammohan KW, Mendell JR (1991). Mononuclear cell analysis of muscle biopsies in prednisone-treated and untreated Duchenne muscular dystrophy. CIDD Study Group. Neurology.

[12] Vandebrouck C, Imbert N, Duport G, Cognard C, Raymond G (1999). The effect of methylprednisolone on intracellular calcium of normal and dystrophic human skeletal muscle cells. Neuroscience letters.

[13] Mendell JR (1989). Randomized, double-blind six-month trial of prednisone in Duchenne’s muscular dystrophy. N Engl J Med.

[14] DeSilva S, Drachman DB, Mellits D, Kuncl RW (1987). Prednisone treatment in Duchenne muscular dystrophy. Long-term benefit. Archives of neurology.

[15] Angelini C (2007). The role of corticosteroids in muscular dystrophy: a critical appraisal. Muscle Nerve.

[16] Heier CR (2013). VBP15, a novel anti-inflammatory and membrane-stabilizer, improves muscular dystrophy without side effects. EMBO molecular medicine.

[17] Zhong Z (2003). L-Glycine: a novel antiinflammatory, immunomodulatory, and cytoprotective agent. Curr Opin Clin Nutr Metab Care.

[18] Hall JC (1998). Glycine. JPEN J Parenter Enteral Nutr.

[19] Jacob, T., Ascher, E., Hingorani, A. & Kallakuri, S. Glycine prevents the induction of apoptosis attributed to mesenteric ischemia/reperfusion injury in a rat model. *Surgery* **134**, 457–466, doi:S0039606003001648 [pii] (2003). 10.1067/s0039-6060(03)00164-8 14555933

[20] Spittler A (1999). Immunomodulatory effects of glycine on LPS-treated monocytes: reduced TNF-alpha production and accelerated IL-10 expression. FASEB J.

[21] Ham DJ (2016). Glycine restores the anabolic response to leucine in a mouse model of acute inflammation. Am J Physiol Endocrinol Metab.

[22] Caldow MK (2016). Glycine supplementation during calorie restriction accelerates fat loss and protects against further muscle loss in obese mice. Clinical nutrition.

[23] Ham DJ, Murphy KT, Chee A, Lynch GS, Koopman R (2014). Glycine administration attenuates skeletal muscle wasting in a mouse model of cancer cachexia. Clinical nutrition.

[24] Gehrig SM, Koopman R, Naim T, Tjoakarfa C, Lynch GS (2010). Making fast-twitch dystrophic muscles bigger protects them from contraction injury and attenuates the dystrophic pathology. Am J Pathol.

[25] Deconinck AE (1997). Utrophin-dystrophin-deficient mice as a model for Duchenne muscular dystrophy. Cell.

[26] Gehrig SM (2012). Hsp72 preserves muscle function and slows progression of severe muscular dystrophy. Nature.

[27] Koopman R (2007). Role of Amino Acids and Peptides in the Molecular Signaling in Skeletal Muscle After Resistance Exercise. Int J Sport Nutr Exerc Metab.

[28] Straub V, Campbell KP (1997). Muscular dystrophies and the dystrophin-glycoprotein complex. Current opinion in neurology.

[29] Pane, M. *et al*. The 6 minute walk test and performance of upper limb in ambulant duchenne muscular dystrophy boys. *PLoS Curr* **6**, 10.1371/currents.md.a93d9904d57dcb08936f2ea89bca6fe6 (2014). 10.1371/currents.md.a93d9904d57dcb08936f2ea89bca6fe6 PMC4208936 25642376

[30] Pane M (2014). 6 Minute walk test in Duchenne MD patients with different mutations: 12 month changes. PLoS One.

[31] Cobos EJ (2012). Inflammation-induced decrease in voluntary wheel running in mice: a nonreflexive test for evaluating inflammatory pain and analgesia. Pain.

[32] Sherwin CM (1998). Voluntary wheel running: a review and novel interpretation. Animal behaviour.

[33] Stevenson GW (2011). Monosodium iodoacetate-induced osteoarthritis produces pain-depressed wheel running in rats: implications for preclinical behavioral assessment of chronic pain. Pharmacology, biochemistry, and behavior.

[34] Kornegay JN (2014). Pharmacologic management of Duchenne muscular dystrophy: target identification and preclinical trials. ILAR J.

[35] Spurney CF (2009). Preclinical drug trials in the mdx mouse: assessment of reliable and sensitive outcome measures. Muscle Nerve.

[36] Koopman R, Caldow MK, Ham DJ, Lynch GS (2017). Glycine metabolism in skeletal muscle: implications for metabolic homeostasis. Curr Opin Clin Nutr Metab Care.

[37] Warren GL (2005). Chemokine receptor CCR2 involvement in skeletal muscle regeneration. FASEB J.

[38] Munoz-Canoves P, Scheele C, Pedersen BK, Serrano AL (2013). Interleukin-6 myokine signaling in skeletal muscle: a double-edged sword?. FEBS J.

[39] Lu H, Huang D, Ransohoff RM, Zhou L (2011). Acute skeletal muscle injury: CCL2 expression by both monocytes and injured muscle is required for repair. FASEB J.

[40] Swiderski K (2014). Tranilast administration reduces fibrosis and improves fatigue resistance in muscles of mdx dystrophic mice. Fibrogenesis & tissue repair.

[41] Webb TI, Lynch JW (2007). Molecular pharmacology of the glycine receptor chloride channel. Curr Pharm Des.

[42] Kennedy TL (2016). BGP-15 Improves Aspects of the Dystrophic Pathology in mdx and dko Mice with Differing Efficacies in Heart and Skeletal Muscle. Am J Pathol.

[43] Weller C (2012). Motor performance of young dystrophic mdx mice treated with long-circulating prednisolone liposomes. Journal of neuroscience research.

[44] Baltgalvis KA, Call JA, Nikas JB, Lowe DA (2009). Effects of prednisolone on skeletal muscle contractility in mdx mice. Muscle Nerve.

[45] Hartel JV, Granchelli JA, Hudecki MS, Pollina CM, Gosselin LE (2001). Impact of prednisone on TGF-beta1 and collagen in diaphragm muscle from mdx mice. Muscle Nerve.

[46] Janssen PM (2014). Prednisolone attenuates improvement of cardiac and skeletal contractile function and histopathology by lisinopril and spironolactone in the mdx mouse model of Duchenne muscular dystrophy. PLoS One.

[47] Keeling RM, Golumbek PT, Streif EM, Connolly AM (2007). Weekly oral prednisolone improves survival and strength in male mdx mice. Muscle Nerve.

[48] Morrison-Nozik A (2015). Glucocorticoids enhance muscle endurance and ameliorate Duchenne muscular dystrophy through a defined metabolic program. Proc Natl Acad Sci USA.

[49] Murphy KT, Chee A, Trieu J, Naim T, Lynch GS (2012). Importance of functional and metabolic impairments in the characterization of the C-26 murine model of cancer cachexia. Disease models & mechanisms.

[50] Schertzer JD, Gehrig SM, Ryall JG, Lynch GS (2007). Modulation of insulin-like growth factor (IGF)-I and IGF-binding protein interactions enhances skeletal muscle regeneration and ameliorates the dystrophic pathology in mdx mice. Am J Pathol.

[51] Gehrig SM, Ryall JG, Schertzer JD, Lynch GS (2008). Insulin-like growth factor-I analogue protects muscles of dystrophic *mdx* mice from contraction-mediated damage. Exp Physiol.

[52] Lynch GS (1997). Contractile properties of diaphragm muscle segments from old mdx and old transgenic mdx mice. The American journal of physiology.

[53] Hoaglin, D. C., Iglewicz, B. & Tukey, J. W. Performance of Some Resistant Rules for Outlier Labeling. *J Am Stat Assoc* **81**, 991–999, doi:10.2307/2289073 (1986).

